# Microwave-Assisted Extraction, Purification, Partial Characterization, and Bioactivity of Polysaccharides from *Panax ginseng*

**DOI:** 10.3390/molecules24081605

**Published:** 2019-04-23

**Authors:** Jing-Li Zhao, Meiping Zhang, Hong-Li Zhou

**Affiliations:** 1 College of Life Science, Jilin Agricultural University, Changchun 130118, China; jinglizhao2005@126.com (J.-L.Z.); meiping.zhang@jlau.edu.cn (M.Z.); 2 Institution of Pharmaceutical and Environmental Technology, Jilin Vocational College of Industry and Technology, Jilin 132013, China; 3 Engineering Research Center for Agricultural Resources and Comprehensive Utilization of Jilin Provence, Jilin Institute of Chemical Technology, Jilin 132022, China

**Keywords:** *Panax ginseng* polysaccharides, microwave-assisted extraction, response surface methodology, structure characterization, bioactivity

## Abstract

Polysaccharides are a main active substance in *Panax ginseng*; however, microwave-assisted extraction used to prepare *P. ginseng* polysaccharides (MPPG) has rarely been reported, and knowledge of the bactericidal activity of *P. ginseng* polysaccharides remains low. Thus, this study was designed to investigate the extraction of *P. ginseng* polysaccharides by using two methods—hot water extraction and microwave-assisted extraction—and compare their chemical composition and structure. In addition, their antibacterial and antioxidant activities were also determined. The data implied that *P. ginseng* polysaccharides extracted by microwave-assisted extraction possessed a higher extraction yield than hot water extraction (WPPG) under optimized conditions, and the actual yields were 41.6% ± 0.09% and 28.5% ± 1.62%, respectively. Moreover, the preliminary characterization of polysaccharides was identified after purification. The WPPG with the molecular weight (Mw) of 2.07 × 10^5^ Da was composed of Man, Rib, Rha, GalA, Glu, Gal, and Arab, and the typical characteristics of polysaccharides were determined by IR spectra. Compared with WPPG, MPPG had a higher Mw, uronic acid content, and Glu content. More importantly, the antioxidant activity of MPPG was higher than WPPG, which was probably ascribed to its highly Mw and abundant uronic acid content. Besides, both of them exhibited high bactericidal activity. These results demonstrate that microwave-assisted extraction is an effective method for obtaining *P. ginseng* polysaccharides, and MPPG could be applied as an antioxidant and antibacterial agent.

## 1. Introduction

Some polysaccharides are polymers of monosaccharide residues attached to each other by glycosidic linkages and belong to macromolecules that are characterized by a structurally various class; these have been widely distributed in higher photosynthetic plants, fungi, algae, etc. [1]. Many natural polysaccharides are highly valuable biomaterials that are characterized by their biocompatibility, low toxicity, and biodegradable properties. As a result, they have become the focus of attention in clinical and experimental research [2]. Meanwhile, it is noteworthy that plant-derived polysaccharides possess various pharmacological properties, including antioxidant, anti-tumor, and immunomodulatory [3,4]. Therefore, it is important to obtain high-activity polysaccharides and explore their potential biological activity in other fields.

To the best of our knowledge, various methods including hot extraction, enzyme extraction, dilute alkaline water extraction, and microwave extraction have been used to obtain these polysaccharides and improve their extraction efficiency. Among these extraction methods, hot extraction is the most convenient and classical method; thus, it is widely used in industry production [5]. However, enzyme extraction, which is commonly used to extract chemical components from herbal medicines, possesses various advantages, including environmental friendliness, lower energy requirements, and high extraction yield. At the same time, its disadvantages, which include higher cost and easy inactivation, are not negligible [6]. The dilute alkaline water extraction method is utilized to extract some acidic polysaccharides or high molecular weight polysaccharides [6], but it can also decrease the extraction rate or change the physicochemical properties of polysaccharides. Compared with these extraction methods, microwave-assisted extraction can enhance the extraction rate more efficiently through facilitating the release of intracellular substances and extract the nature-based bioactive constituents, such as polysaccharides that are present in the cell walls [7]. It is also more environmental friendly due to the reduced consumption of solvents and energy [8,9,10]. However, many research studies have indicated that extraction methods can significantly affect the yield of polysaccharides, as well as their physicochemical properties and biological activities [11,12]; that is to say, the effects on the extraction yield, structural characteristics, and biological characteristics of the polysaccharides prepared by different methods should not be neglected. In order to improve the extraction yield of polysaccharides, the optimal extraction parameters are determined by response surface methodology (RSM). RSM includes the statistical optimization of various factors such as Box–Behnken design (BBD), Doehlert design (DD), and Central composite design (CCD), and has been widely employed in various extraction processes, as it can simulate a true limit state surface, validate the statistical significance of the independent variables, and obtain the maximal extraction yield under the optimum conditions [13]. 

*Panax ginseng* C. A. Meyer (ginseng) (Family Araliaceae) is a species of a slow-growing perennial plant with fleshy roots that is mainly grown in the northeast of China, and has been widely used for thousands of years in China and other Asian countries for medicinal treatment, especially for cancer, diabetes, and heart problems [14,15]. Polysaccharides are the most active components of *P. ginseng*, which also possesses various medicinal properties, including anti-tumor, immunomodulation, and antioxidative activities [16]. Many research studies have been conducted on *P. ginseng* polysaccharides since 1996, but among these reports, most of the polysaccharides are extracted from *P. ginseng* by using hot water methods, which is also due to *P. ginseng* polysaccharides mainly being water-soluble polysaccharides [17,18,19]. Among these previous studies, Luo and Fang [18] reported that using purified hot water extraction to prepare *P. ginseng* polysaccharides (WPPG) exhibited a strong antioxidant activity with an average molecular weight (Mw) of 3 × 10^5^ Da and 4 × 10^5^ Da, respectively, which was mainly comprised of glucose. However, to date, there has been little information on the specifically physiochemical characteristics of using microwave-assisted extraction to prepare *P. ginseng* polysaccharides (MPPG). In addition, the antibacterial activity of *P. ginseng* polysaccharides remained unclear. Therefore, our paper aims to determine whether the microwave-assisted extraction process is optimized with RSM. Furthermore, the physiochemical characteristics of the purified WPPG and MPPG methods, including their infrared spectrum (IR), molecular weight (Mw), and monosaccharide composition are compared. More importantly, the biological activities of polysaccharides extracted by hot water extraction and microwave extraction are respectively evaluated, as demonstrated by linking these activities with their physiochemical characteristics.

## 2. Results and Discussions

### 2.1. Single-Factor Experiment

**Figure 1:**
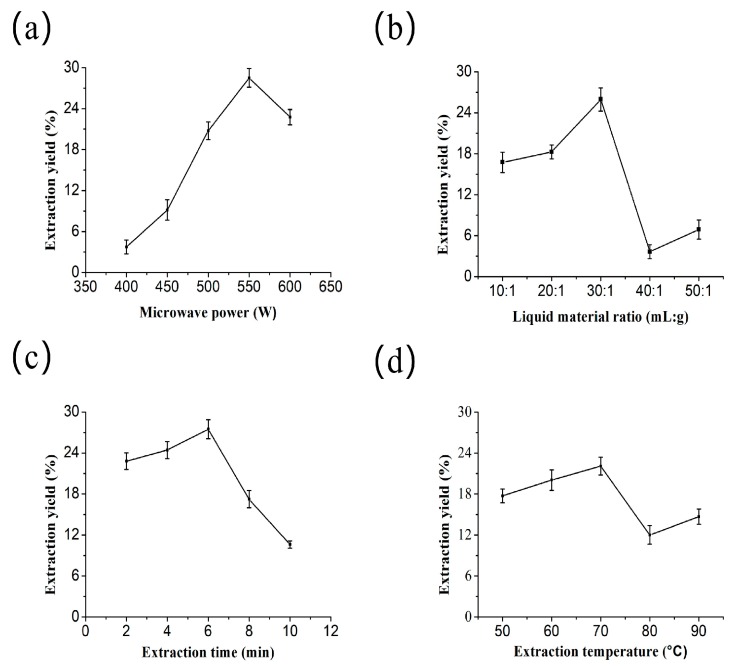
Effects of microwave power (**a**), liquid-to-material ratio (**b**), extraction time (**c**), and extraction temperature (**d**) on the extraction yield of microwave-assisted extraction used to prepare *P. ginseng* polysaccharides (MPPG). Data were expressed as the means ± SD (*n* = 3).

To the best of our knowledge, the extraction parameters, including microwave power, liquid-to-solid ratio, extraction time, and extraction temperature are important, since they can affect the extraction rate of polysaccharides [10]. Figure 1a shows that the extraction yield significantly increased the microwave power from 400 W to 550 W, which reached a maximum yield at 550 W. However, the extraction yield was ultimately decreased with the increase of microwave power after 550 W, which might be due to the glycosidic linkage of MPPG being damaged by the higher microwave power, and subsequently leading to the degradation of the polysaccharides [20]. Moreover, the enhanced microwave power will decrease the antioxidant activity of the polysaccharides, which is mostly due to the biological activity of polysaccharides being decreased due to the rising temperature [10]. Therefore, 550 W was considered to be the optimal microwave power in this experiment. 

Next, the extraction yield of MPPG significantly increased during the liquid-to-solid ratio of approximately 10:1 to 30:1 mL/g, and reached a maximum yield at 30:1 mL/g. As the liquid-to-solid ratio further increased, the extraction yield continuously decreased (from approximately 30:1 to 40:1 mL/g) (Figure 1b), which might be due a higher liquid-to-solid ratio possibly leading to a lower density and viscosity, thereby facilitating the dilution of polysaccharides in the solvent [21]. Furthermore, it can cause a higher processing cost. Accordingly, the optimal liquid-to-solid ratio was 30:1 mL/g. 

Meanwhile, the effect of extraction time on the yield of MPPG in Figure 1c showed that the extraction yield increased within 2 min to 6 min, and reached a maximum yield at 6 min. The results showed a downward trend after 6 min, which was probably caused by partial degradation [22]. Simply put, the reason may be that with the increasing time, the polysaccharides in the solvent are affected by the constant temperature [23]. Thus, based on these results, the optimum extraction time was 6 min. 

Finally, with the extraction temperature rising from 50 °C to 70 °C, the MPPG extraction yield slightly increased (Figure 1d) and decreased after 70 °C, which indicated that the rising temperature may destroy the stability of the polysaccharides’ structure [22], or change the properties of the polysaccharides [24]. Thus, the extraction temperature at 70 °C was favorable for producing the polysaccharides.

Indeed, the optimal extraction condition will not only increase the extraction yield, but also decrease the cost through reducing the required time, energy, solvent, and materials. Therefore, the optimal extraction parameters were selected for further optimization experiments in this work.

### 2.2. Model Fitting and Statistical Analysis

Based on the results of single-factor experiments, the design matrix and actual results of RSM experiments were shown in Table 1. As shown in Table 1, the MPPG extraction yield ranged from 17.1% to 42.4%, and the maximum extraction yield (42.4%) was obtained under the following conditions: X_1_ = 550 W, X_2_ = 30: 1 mL/g, X_3_ = 6 min, and X_4_ = 70 °C. These results were fitted with the following second-order polynomial equation in terms of coded values:

Y = 40.57 + 2.02X_1_ + 4.10X_2_ − 0.87X_3_ − 2.20X_4_ − 3.39X_1_X_2_ − 0.91X_1_X_3_ + 4.84X_1_X_4_ + 0.26X_2_X_3_ + 1.88X_2_X_4_ − 2.06X_3_X_4_ − 4.69X_1_^2^ − 5.50X_2_^2^ − 4.81X_3_^2^ − 4.09X_4_^2^

Here, Y is the extraction yield (%). X_1_, X_2_, X_3_, and X_4_ are the coded values of the microwave power, liquid-to-solid ratio, extraction time, and extraction temperature, respectively.

### 2.3. Analysis of Response Surface

Design-Expert is software that predicts the extraction yield by exploring the relationship between the independent and dependent variables; it has been widely used by other researchers for the extraction process. In this paper, the MPPG yield was affected by the four above-mentioned variables. In all of the figures, the relationship between the extraction yield of MPPG and any two independent variables (other factors were set at level zero) was shown, as evidenced by the shapes of the contour plots, whether circular or elliptical. Briefly, the interactions between the relevant parameters with a circular shape were negligible, but, when the elliptical contour plot was observed, the interaction between the relevant parameters was significant. 

**Figure 2:**
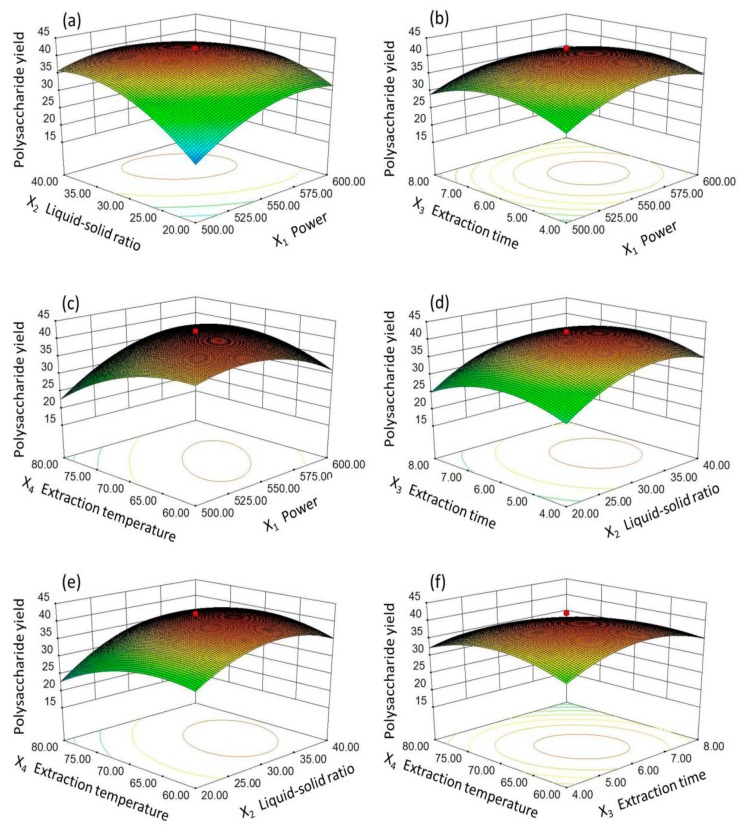
Response surface plots (3D) for the microwave-assisted extraction of MPPG. (**a**) Liquid-to-solid ratio versus microwave power; (**b**) Extraction time versus microwave power; (**c**) Extraction temperature versus microwave power; (**d**) Liquid-to-solid ratio versus extraction time; (**e**) Extraction temperature versus liquid-to-solid ratio; and (**f**) Extraction temperature versus extraction time.

**Figure 3:**
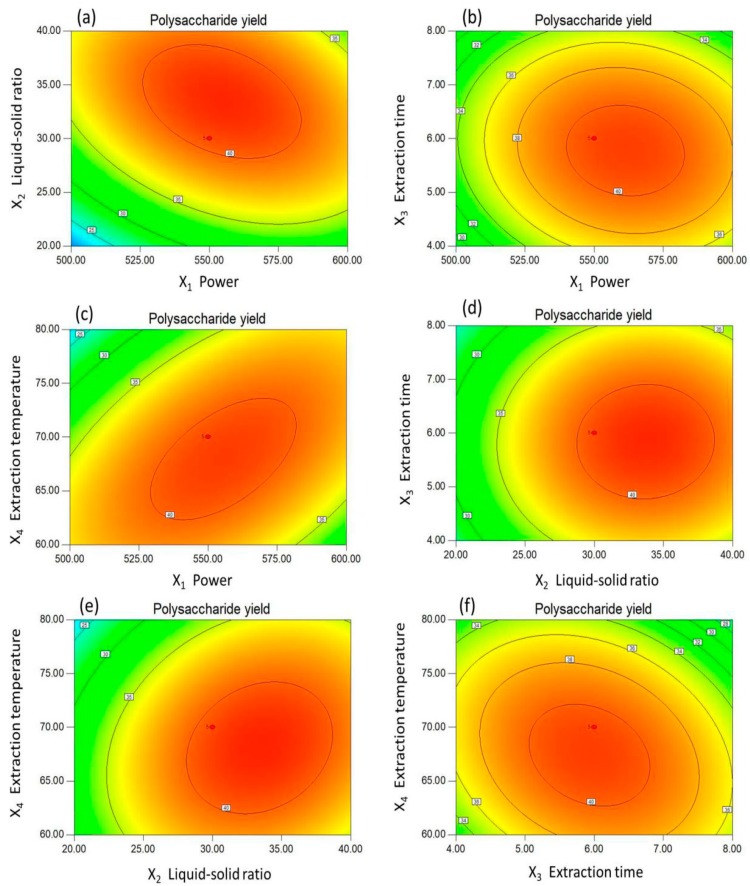
Response surface plots (2D) for the microwave-assisted extraction of MPPG. (**a**) Liquid-to-solid ratio versus microwave power; (**b**) Extraction time versus microwave power; (**c**) Extraction temperature versus microwave power; (**d**) Liquid-to-solid ratio versus extraction time; (**e**) Extraction temperature versus liquid-to-solid ratio; and (**f**) Extraction temperature versus extraction time.

For example, when the three-dimensional (3D) response surface plot and the contour plot were plotted for the yield of MPPG with varying liquid-to-solid ratios and microwave power at a fixed number (0 level) of other independent variables (extraction time and extraction temperature) (Figure 2a and Figure 3a), the yield first increased as the liquid-to-solid ratio was increased from 20 to 30 mL/g, and reached a peak value significantly at a liquid-to-solid ratio of 30 mL/g. Afterwards, the yield slightly decreased. The yield also increased as the microwave power increased from 500 to 550 W, but, for more than 550 W, the yield then decreased. In addition, as shown in Figure 3a, the shapes of the contour plots was elliptical; that is to say, the interaction between the corresponding variables had a significant effect on the extraction yield. The interaction between any other two extraction parameters on the yield of MPPG was similar (Figure 2b–f and Figure 3b–f).

In addition, the MPPG yield was sensitive to the alteration of the variables, and it could be observed that the yield of MPPG slightly increased along with the increasing value of each factor within a certain range. However, when the extraction yield reached the maximum value, the yield could not increase, even though each factor continued to increase. Furthermore, according to ANOVA (Table 2), the order of the effect of the four independent variables on the yield of polysaccharides was X_2_ > X_4_ > X_1_ > X_3_; in particular, X_2_ (the liquid-to-solid ratio) demonstrated a significant difference among the parameters (*p* < 0.01). Moreover, X_1_ (microwave power) and X_4_ (extraction temperature) had greater correlation (*p* < 0.01) on the yield of polysaccharides.

### 2.4. Verification of the Predictive Model

Based on the results of the RSM, the optimal extraction parameters were obtained as follows: 550 W (microwave power), 30 mL/g (liquid-to-solid ratio), 6 min (extraction time), and 70 °C (extraction temperature), and the theoretical extraction yield was 42.4%. To determine the accuracy of the model equation, a verification experiment was carried out under optimal conditions, and the obtained extraction yield was at 41.6 ± 0.09% (*n* = 3), suggesting that the regression model is applied for the microwave extraction of MPPG.

### 2.5. Extraction Yield of Hot Water on P. Ginseng Polysaccharides

The extraction yield of WPPG was 28.5% ± 1.62% (*n* = 3) (Table 3). Compared with microwave extraction (41.6%), the extraction yield of WPPG was lower, which indicated that microwave-assisted extraction could serve as an effective method for extracting *P. ginseng* polysaccharides. 

### 2.6. Purification of Crude MPPG and WPPG

After the purification of MPPG and WPPG by diethyl amino ethyl (DEAE) cellulose, the content of total carbohydrates reached 81.7 ± 3.02% and 84.8 ± 2.98%, respectively. The residual protein levels of MPPG and WPPG were 1.67 ± 0.19% and 1.73 ± 0.36%, respectively. The content of uronic acid was 10.5 ± 1.77% and 8.5 ± 1.54%, respectively. In addition, the contents of ginsenoside in purified polysaccharides were not present.

### 2.7. Analysis of UV and IR

**Figure 4:**
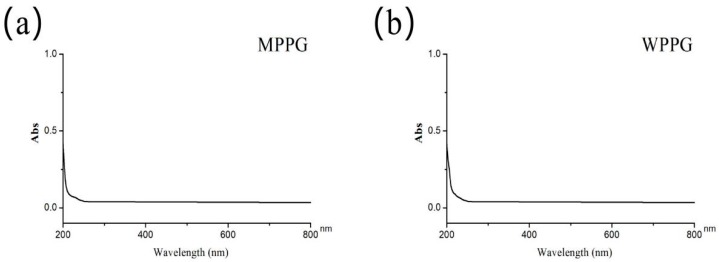
UV spectrum of MPPG (**a**) and WPPG (**b**).

The UV scanning spectra of MPPG (a) and WPPG (b) was observed in Figure 4. No nucleic acids and proteins appeared in the purified polysaccharides, which was evidenced by no significant absorption peaks at 260 nm and 280 nm, which was also consistent with the qualitative analysis.

**Figure 5:**
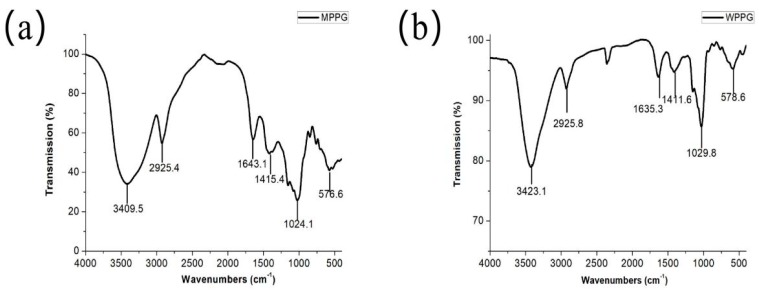
IR spectrum of MPPG (**a**) and hot water extraction used to prepare *P. ginseng* polysaccharides (WPPG) (**b**).

Meanwhile, as shown in Figure 5a,b, both of the polysaccharides possessed similar absorption patterns, suggesting that microwave extraction had insignificant changes on the structure of polysaccharides. Specifically, the absorption peaks at around 3423 cm^−1^ corresponded to the O–H stretching vibration, and the weak peaks at 2925 cm^−1^ were characteristic of C–H, which existed in –CH2 and –CH3. The absorption peaks at approximately 1643 cm^−1^ and 1411 cm^−1^ were attributed to the asymmetric stretching vibration of the absorption peak C=O and symmetric stretching vibrations of COO^−^ [25], respectively, revealing the presence of galacturonic acid in pectin polysaccharides [26]. Two peaks at 1024.1 cm^−1^ and 1029.8 cm^−1^ indicated the presence of a pyranose form. Interestingly, these obtained characteristic peaks were in agreement with previous study [27], but the extraction yield indicated a significant difference. Zhang et al. had reported that the optimal extraction conditions were as follows: 600 W (microwave power), 50 mL/g (liquid-to-solid ratio), 6 min (extraction time), and the actual extraction yield was 30%. In our paper, the extraction temperature is an important factor that has received our attention, because a high temperature can cause the decay of the polysaccharide, reducing its pharmacological activity [9]. Therefore, the extraction temperature was determined in Section 2.1 (d). Indeed, the actual extraction yield was 41.6% under optimal condition, which had demonstrated that the extraction temperature should not be negligible.

### 2.8. Analysis of Monosaccharide Composition

Compared with the retention time of the standard mixture, the monosaccharide composition is shown in Table 4. The main monosaccharides of MPPG were Man, Rib, Rha, GluA, GalA, Glu, Gal, and Arab at a molar ratio of 3.94:4.55:1.85:1.0:1.43:141.42:2.7:5.15, respectively. The main monosaccharides of WPPG were Man, Rib, Rha, GalA, Glu, Gal, and Arab at a molar ratio of 3.75:3.42:1.09:1.0:67.6:1.56:1.1, respectively. These results illustrated that Glu might be the main backbone of the structure, which was consistent with previous study [18]. 

### 2.9. Analysis of Molecule Weight

HPSEC was an effective method for determining the Mw of polysaccharides. As shown in Table 4, the WPPG and MPPG both had two peaks. According to the calibration curve, the Mw of the WPPG peaks were 527763 Da and 402 Da, and the corresponding relative contents of the WPPG peaks were 39.1% and 60.1%, thereby leading to an average Mw was 2.07 × 10^5^ Da. The Mw of the MPPG peaks were 1,271,520 Da and 411 Da, and the contents were 29% and 71%, respectively. So, the average molecular weight of MPPG was 3.69 × 10^5^ Da.

### 2.10. Analysis of Antibacterial Activity

As shown in the Table 5, *P. ginseng* polysaccharides possess highly inhibitory effects on these bacterial strains (*S. aureus*, *E. coli*, *B. pumilus*, and *B. subtilis*), especially on *E. coli*. More specifically, the minimal inhibitory concentrations (MICs) of MPPG on the bacterial strains was 0.25 mg/mL, 0.025 mg/mL, 0.01 mg/mL, and 0.5 mg/mL, respectively. Furthermore, the MIC of WPPG was higher than MPPG, which also represented a lower bactericidal activity, as evidenced by the values of 0.5 mg/mL, 0.05 mg/mL, 0.05 mg/mL, and 0.5 mg/mL. 

Although we first provided the evidence that *P. ginseng* polysaccharides could prevent bacterial visible growth, the exact mechanisms of bactericidal activity by *P. ginseng* polysaccharides remain unclear. Recently, Zhang et al. [28] had proposed that Cordyceps cicadae polysaccharides possessed bactericidal activity by damaging the cell wall and cell membranes, which increased the cell permeability, thereby leading to the structural lesions and release of cell components, and especially damaging the membrane protein of *E coli*. Indeed, protein as part of a bacterial structure plays a variety of biochemical reactions, including catalytic, protein synthesis, and bacteria metabolism. Therefore, the mechanism of bactericidal activity will become the focus of the attention in our further study. 

### 2.11. Analysis of Antioxidant Activities

**Figure 6:**
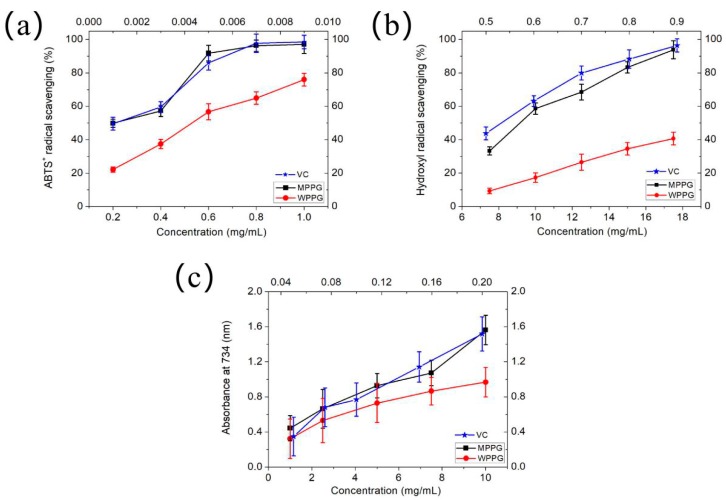
In vitro antioxidant activity of MPPG and WPPG by ABTS radical scavenging assay (**a**), hydroxyl radical scavenging activity (**b**), and total reducing power assay (**c**). Vitamin C (VC) was used as the positive control. Values represent mean ± SD (*n* = 3).

The ABTS assay is a common test to evaluate antioxidant capacity that has been widely applied in plant extracts or food products, especially as evidenced by reflecting the antioxidant effect of the purified polysaccharides [29]. In this paper, the radical scavenging activities of the polysaccharides and vitamin C (VC) were observed in Figure 6a. The scavenging activities of VC, MPPG, and WPPG correlated well with increasing concentrations. When the concentration reached 1.0 mg/mL, the scavenging activities of MPPG was slightly lower than VC (97.09% versus 98.5%) and stronger than WPPG (97.09% versus 75.99%). Furthermore, the IC50 values were 0.001 (VC), 0.236 (MPPG), and 0.507 (WPPG) mg/mL, respectively, indicating that MPPG possessed higher scavenging activities of ABTS than WPPG.

Hydroxyl radicals, among the reactive oxygen, are considered to be highly effective in biological tissues, and especially the main factor for the oxidative injury of most biomolecules, including amino acids, proteins, and DNA in cells [30]. Thus, scavenging hydroxyl radicals is crucial for antioxidant defense. The hydroxyl radical scavenging effect of WPPG and MPPG was shown in Figure 6b. The hydroxyl radical scavenging activity of MPPG and WPPG both increased in a concentration-dependent manner in the range of approximately 7.5 to 17.5 mg/mL. Although the scavenging activity of MPPG was slightly lower than VC, MPPG exhibited a stronger ability than WPPG. Furthermore, the values of IC50 were 9.271 mg/mL (MPPG), 20.16 mg/mL (WPPG), and 0.533 (VC) mg/mL, respectively. A previous study had shown that the hydroxyl radical scavenging effect was attributed to the structure of polysaccharides that could provide hydrogen, combining with multiple radicals and forming stable radicals to terminate the radical chain reaction [31]. 

The assay of reducing power, which is a method to convert the oxidized form of iron (Fe^3+^) to its reduced form (Fe^2+^), is commonly used to evaluate antioxidant potential. Therefore, the ability of the reducing power that is used to assess the antioxidant activity of drugs is important. The values of the total reducing power are shown in Figure 6c. It is noteworthy that the absorbance of MPPG, WPPG, and VC increased as the concentration increased, indicating a concentration-dependent manner. MPPG exhibited a higher antioxidant activity than WPPG at the same concentration, while exhibiting a lower antioxidant activity than VC. The results demonstrated that MPPG can enhance the antioxidant activity, which is probably due to the presence of electron-donating groups or a hydrogen atom, thereby reacting with a free radical to stabilize and block free radical chain reactions [32].

Generally, extraction is the first step to obtain biological macromolecules from natural products, and more importantly, the structure and bioactivities of biological macromolecules are affected by extraction methods. Therefore, it is important to explore the relationship between the extraction method and bioactivities. Previous reports had indicated that various extraction methods led the different antioxidant activities [33]. In this paper, *P. ginseng* polysaccharides extracted by hot water and microwave extraction exhibited significantly different antioxidant activity abilities, especially at the highest concentration (ABTS: 97.09% versus 75.79%; hydroxyl radical: 93.8% versus 40.7%; Total reducing power: 1.562 versus 0.967). Although there has not been a consensus regarding the exact antioxidant mechanism of polysaccharides up to now, the physicochemical properties, including the Mw and chemical composition influencing the activities, has been reported [34,35]. It has been suggested that polysaccharides with lower Mw have higher antioxidant activities; however, Sheng and Sun [36] reported that the polysaccharides extracted from *Athyrium multidentatum* (Doll.) Ching possessed stronger reducing power due to the higher Mw. The Mw of MPPG and WPPG (3.69 × 10^5^ Da versus 2.07 × 10^5^ Da) was determined in this paper, and the total reducing power of MPPG was higher than WPPG, which was consistent with the above study. Therefore, the effect of Mw on antioxidant activities is still unclear. 

In addition, monosaccharide composition as one of the physicochemical properties of polysaccharides also affects the antioxidant activities. Zhang et al. [35] reported that the polysaccharides exhibited different reducing powers, which might be due to the Gal content. In our paper, the content of Gal polysaccharides was at a molar ratio of 2.70 (MPPG) and 1.56 (WPPG), respectively, indicating that the reducing power of MPPG was higher than WPPG, which was consistent with the above study. In addition, many research studies have demonstrated that the higher activity was attributed to the larger uronic acid content. Gao et al. [37] had reported that polysaccharides extracted from *Laminaria japonica* exhibited higher antioxidant activity, which was due to an abundant amount of uronic acid. Therefore, we made a hypothesis that the possible mechanism of antioxidant activities of MPPG might be due to the higher uronic acid content. However, the antioxidant activities of polysaccharides are not influenced by a single factor, but rather a combination of several factors. Therefore, studies on the structure–function relationship of plant polysaccharides are in progress, and more details will be reported in the future.

## 3. Materials and Methods

### 3.1. Materials and Chemicals

Four-year-old roots of *P. ginseng* were collected from Changbai Mountain district, Jilin, China, and were authenticated by Prof. Guangshu Wang, School of Pharmaceutical Sciences, Jilin University, Changchun, China.

Distilled water was purchased from Hangzhou Wahaha Group Co., Ltd. (Hangzhou, China); 1, phenanthroline, ferrous sulfate (FeSO_4_), potassium ferricyanide, trichloroacetic acid, ferric chloride (FeCl_3_), ferrozine, vitamin C (VC), and ethylene diamine tetraacetic acid (EDTA) were from Macklin Biochemical Co., Ltd. (Shanghai, China); trifluoroacetic acid (TFA), hydroxylamine hydrochloride, pyridine, acetic anhydride, chloroform, n-butanol, and ethanol were from Sigma Aldrich Chemical Co., Ltd. (St. Louis, MO, USA); DEAE (diethyl amino ethyl) cellulose was from Yuanye Bio-Technology Co., Ltd. (Shanghai, China); Standard sugars including mannose, ribose, rhamnose, glucuronic acid, galacturonic acid, glucose, galactose, and arabinose were from Sino-pharm Chemical Reagent Co., Ltd. (Shanghai, China); T-series Dextran standards including T-10, T-40, T-70, T-100, and T-500 kDa were from the National Institutes of Food and Drug Control (Beijing, China). All of the reagents used in this study were of the highest quality available from commercial vendors and were of analytical grade. 

### 3.2. Microwave-Assisted Extraction Process of P. Ginseng Polysaccharides

*P. ginseng* polysaccharides were obtained by using microwave-assisted extraction based on the reported method with some modifications [38]. In brief, the roots of *P. ginseng* were collected, washed, crushed in a mortar, and degreased with petroleum ether to form the final ginseng juice and pomace. Next, 10 g of mixed residue was added into distilled water at a different liquid-to-solid ratio, and put into a microwave extraction apparatus (MAS-II Plus, Sineo Microwave, Shanghai, China). When the extraction of polysaccharides was accomplished, the supernatant was concentrated to reach 20 mL by rotary evaporation under reduced pressure. The concentrated solution was placed in a beaker, followed by mixing with four volumes of 95% ethanol to precipitate crude MPPG at 4 °C overnight. The crude MPPG was finally obtained by centrifugation and grounded into powders after freeze-drying.

Besides, the total polysaccharide content was determined by phenol sulfuric acid assay at 490 nm using glucose as a standard [39]. The standard curve and *P. ginseng* polysaccharides extraction yield were expressed as follows:

A = 7.08C + 0.0601 (R^2^ = 0.99904), (1)

Here, A represents the absorbance; C represents the polysaccharides content (mg/mL); and the linear range was from 0.06 to 0.14 mg/mL.

*P. ginseng* polysaccharides yield (%) = C × V × 10^−3^/M × 100, (2)

where C represents the polysaccharides content (mg/mL); V represents the volume of the filtrate (mL); and M represents the weight of *P. ginseng* juice and pomace (g).

### 3.3. Single-Factor Experimental Design for P. Ginseng Polysaccharides Extraction

The main parameters including the microwave power, liquid-to-solid ratio, extraction time, and extraction temperature affect the extraction yield in the microwave-assisted extraction process of *P. ginseng* polysaccharides. To explore the impact of various factors on the polysaccharides extraction yield, the extraction rate of crude *P. ginseng* polysaccharides, which was used as an indicator, was determined by using a single-factor experimental design. The single factors included microwave power (400–600 W), liquid-to-solid ratio (10:1–50:1 mL/g), extraction time (2–10 min), and extraction temperature (50–90 °C). In addition, one factor was changed when the other factors were kept constant in each experiment. All of the experiments were carried out in triplicate.

### 3.4. Experimental Design for Optimization

According to the results of preliminary single-factor tests, the extraction conditions of *P. ginseng* polysaccharides were then optimized by using RSM. A four-variable and three-level CCD containing 21 runs was applied at the center point using extraction yield as the response value (Table 6). 

Regression analysis was applied for the experimental data and fitted to the following second-order polynomial equation:

$$Y=\beta_{0}+\sum_{i=1}^{4}\beta_{i}X_{i}+\sum_{i=1}^{4}\beta_{ii}X_{i}^{2}+\sum_{i=1}^{3}\sum_{j=i+1}^{4}\beta_{ij}X_{i}X_{j}$$

where *Y* represents the response function; *β*_0_ represents the intercept; *β_i_*, *β_ii_* and *β_ij_* represent the coefficients of linear, quadratic, and interactive terms, respectively; and *X_i_* and *X_j_* represent the coded independent variables. Design-Expert software 8.0.6.1 (Stat-Ease, Minneapolis, MN, USA) was used to estimate the response of the independent variables, and was applied to the calculation of the predicted data. Next, each test was repeated three times, and a value of *p* < 0.05 was considered to be statistically significant.

### 3.5. Hot Water Extraction of P. Ginseng Polysaccharides

*P. ginseng* polysaccharides were extracted with a traditional hot water method in a water bath, and the optimal extraction conditions were finally chosen as follows: the extraction temperature was 90 °C, the extraction time was 5 h, and the liquid-to-solid ratio was 1:40 (g/mL). After extraction, the polysaccharides were obtained through using the method outlined in Section 3.2. 

### 3.6. Purification and Isolation of P. Ginseng Polysaccharides

The protein in the concentrated solution was removed by using Sevag reagent (chloroform and n-butanol at a ratio of 4:1) [40]. After removing the Sevag reagent, the solution was dialyzed (MD10, Viskase, Darien, IL, USA) in distilled water for 72 h and precipitated again by 80% ethanol. The precipitate was redissolved in distilled water and subjected to a DEAE cellulose column. Briefly, the polysaccharides were applied to a DEAE cellulose column (2.6 × 30 cm) and then eluted with 0.5 M of NaCl at a flow rate of 1.0 mL/min (10 mL/tube). The major fractions of purified WPPG and MPPG were finally collected using a fraction collector, and the compounds were detected by the phenol sulfuric acid method [39]. 

### 3.7. Characterization of Physicochemical Properties of MPPG and WPPG

#### 3.7.1. Ultraviolet Spectrum Analysis

The solution of purified MPPG and WPPG prepared at concentration of 1 mg/mL was determined by a UV-vis spectrophotometer (L5S, INESA Analytical Instrument Co. Ltd., Shanghai, China) within 200 nm and 800 nm [41].

#### 3.7.2. Infrared Spectrum Analysis

The purified WPPG and MPPG were ground with KBr, and then pressed into a pellet. The fractions of polysaccharides were finally determined by using an FTIR-650 Fourier transform infrared spectrophotometer (Gangdong Sci. & Tech. Development Co., Ltd, Tianjin, China) within the range of approximately 4000 to 400 cm^−1^ [42].

#### 3.7.3. Molecular Weights Analysis

The Mws of purified WPPG and MPPG were estimated by using a high-performance size-exclusion chromatograph attached to a refractive index detector (RID, RI2000, A, Schambeck SFD GmbH, Bad Honnef, Germany) [43]. The sample solution (20 μL) was injected into a Shodex sugar KS-804 column (8.0 mm × 300 mm), and then analyzed by using HPLC (Elite P230IIHPLC, Elite Analytical Instruments Co. Ltd., Dalian, China). The performance conditions were as follows: a column temperature of 50 °C, a flow rate of 1.0 mL/min, an RID temperature of 35 °C; and a total run rate of 30 min. After that, the different molecular weights of dextran standards were used for the calibration curve.

#### 3.7.4. Monosaccharide Composition Analysis

The monosaccharide composition of purified WPPG and MPPG was determined by gas chromatography (GC) with some modifications [44]. Briefly, 20 milligrams of purified polysaccharides were hydrolyzed to monosaccharide by 2 mL of 2M TFA into a reactor, and kept at 120 °C for 3 h. After removing the residual TFA, the final products were obtained after a series of derivatization. Then, 10 μL of samples were injected into an Ultimate 3000 high-performance liquid chromatograph (HPLC, Thermo, Waltham, MA, USA) equipped with a Supersil ODS2 column (5 m, 4.6 × 250 mm^2^) and an Ultimate 3000 diode array detector (DAD, Thermo). The chromatographic conditions were performed according to the above report. Different monosaccharides were used as standards (Mannose, Ribose, Rhamnose, Glucuronic acid, Galacturonic acid, Glucose, Galactose, Arabinose). The area normalization method was used to calculate the molar ratio of monosaccharide in polysaccharides.

### 3.8. Antibacterial Activity Assay

The minimum inhibitory concentration (MIC) was also considered as the lowest concentration that prevents bacterial visible growth. The bacterial strains, which included *Staphylococcus aureus*, *Escherichia coli*, *Bacillus pumilus*, and *Bacillus subtilis*, were detected according to the broth microdilution assay reported by Jones [45] with some modifications. Simply put, the concentration in a 96-well plate was reduced, and the MPPG and WPPG were diluted into the range of 0.78 to 200 mg/mL. Next, the standard suspension of each bacterial strain (10^8^ CFU·L^−1^) was added into each well. The final concentration of dimethyl sulfoxide (DMSO), which was applied to dissolve the precipitate, reached 20% (*v*/*v*) in each well. All of the assays were repeatedly performed in triplicate. 

### 3.9. In Vitro Antioxidant Activity Assay 

#### 3.9.1. Assay for the ABTS^+^ Radical Scavenging Activity 

The ABTS radical cation (ABTS^+^) scavenging assay was performed as described in the previous study [46] with slight modifications. Simply put, after ABTS^+^ solution was mixed with potassium persulfate, the mixture was stored in darkness to prepare the final ABTS stock solution. The final ABTS stock solution was diluted with ethanol when used for analysis. The sample solution of MPPG and WPPG (prepared at 0.2 mg/mL, 0.4 mg/mL, 0.6 mg/mL, 0.8 mg/mL, and 1.0 mg/mL, respectively) were mixed with 3 mL of ABTS^+^ working solution, followed by incubation for 60 min and the determination of its absorbance at 734 nm (A_1_). The ABTS^+^ working solution was replaced with distilled water, and the absorbance (A_2_) was determined. The blank solution of the sample was determined (A_0_). VC was served as positive control. The ABTS^+^ radical scavenging rate was calculated by Equation (3). Three replicates contributed to each test sample.

ABTS radical-scavenging rate (%) = [1 − (A_1_ − A_2_)/A_0_] × 100, (3)

#### 3.9.2. Assay for the Hydroxyl Radical Scavenging Activity

Hydroxyl radical scavenging was assayed according to the method previously reported by You et al. [47] with modifications. Briefly, 20 μL of purified MPPG and WPPG solution (prepared at 7.5 mg/mL, 10.0 mg/mL, 12.5 mg/mL, 15.0 mg/mL, and 17.0 mg/mL, respectively) were mixed with phenanthroline and PBS (pH = 7.4); then, 20 μL of 0.75 mmoL/L FeSO_4_ was added. After that, the final solution was prepared by adding 20 μL of 0.12% H_2_O_2_, followed by incubation for 60 min at 37 °C; then, its absorbance was determined at 536 nm (As). The system without H_2_O_2_ was used as normal control (Ac), and the system without tested samples was used as the blank solution (A_0_), respectively. VC was served as positive control. The scavenging activity of the hydroxyl radical was calculated by the following Equation (4). Three replicates were applied for each test sample.

Hydroxyl radical-scavenging rate (%) = (As − A_0_) × 100/ (Ac − A_0_), (4)

#### 3.9.3. Assay for the Total Reducing Power Assay

The total reducing power assay was determined according to the method [48] with slight modifications. Briefly, 10 μL of MPPG and WPPG solution (prepared at 1.0 mg/mL, 2.5 mg/mL, 5.0 mg/mL, 7.5 mg/mL, and 10.0 mg/mL, respectively), was mixed with 25 μL of PBS buffer (pH 6.6) and 25 μL of 1% potassium ferricyanide solution, followed by incubation for 20 min at 50 °C. After being cooled and adding 25 μL of 10% trichloroacetic acid solution, the mixture was centrifuged at 3000 rpm for 10 min. Finally, 2.5 mL of supernatant was mixed with 2.5 mL of distilled water and 0.5 mL of 0.1% FeCl_3_ solution, and its absorbance at 700 nm was determined. VC served as positive control. Three replicates were carried out for each test sample.

## 4. Conclusions

Taken together, we extracted polysaccharides from *P. ginseng* by different methods, including microwave-assisted extraction and hot water extraction; then, we partially characterized its chemical composition and structure, and determined its biological activity. Meanwhile, the purpose of this study was to determine the changes in the structural and antioxidant activity of polysaccharides extracted by different methods. The results demonstrated that the antioxidant activity of MPPG was stronger than WPPG, which might be due to the higher Mw, higher Gal content, and higher uronic acid content. Although the structural characteristics, including types of glycosidic linkage and conformation of MPPG, is also needed to investigate in an exhaustive investigation, the findings generated in this study had indicated that the selection of the appropriate extraction method should be considered when exploring ways to obtain polysaccharides with enhanced bioactivity. In addition, we first explored that *P. ginseng* polysaccharides possessed a higher antibacterial activity, as evidenced by the lower MIC. Herein, MPPG could be used as a natural antioxidant and antibacterial agent in the functional food fields and pharmaceutical industries.

## Figures and Tables

**Table 1:** Central composite design and observed responses.

Run	Independent Variable				Extraction Yield (%)
	X_1_	X_2_	X_3_	X_4_	
1	600 (1)	40:1 (1)	8 (1)	60 (−1)	20.9
2	600 (1)	40:1 (1)	4 (−1)	60 (−1)	19.9
3	600 (1)	20:1 (−1)	8 (1)	80 (1)	20.1
4	500 (−1)	40:1 (1)	4 (−1)	80 (1)	24.3
5	600 (1)	20:1 (−1)	4 (−1)	80 (1)	28.4
6	500 (−1)	20:1 (−1)	8 (1)	60 (−1)	23.4
7	500 (−1)	40:1 (1)	8 (1)	80 (1)	20.7
8	500 (−1)	20:1 (−1)	4 (−1)	60 (−1)	19.8
9	465.91	30:1 (0)	6 (0)	70 (0)	22.9
10	634.09	30:1 (0)	6 (0)	70 (0)	29.7
11	550 (0)	13.18	6 (0)	70 (0)	17.1
12	550 (0)	46.82	6 (0)	70 (0)	30.9
13	550 (0)	30:1 (0)	2.64	70 (0)	27.3
14	550 (0)	30:1 (0)	9.36	70 (0)	24.6
15	550 (0)	30:1 (0)	6 (0)	53.18	31.7
16	550 (0)	30:1 (0)	6 (0)	86.82	24.3
17	550 (0)	30:1 (0)	6 (0)	70 (0)	41.9
18	550 (0)	30:1 (0)	6 (0)	70 (0)	42.4
19	550 (0)	30:1 (0)	6 (0)	70 (0)	40.34
20	550 (0)	30:1 (0)	6 (0)	70 (0)	40.45
21	550 (0)	30:1 (0)	6 (0)	70 (0)	40.12

By the analysis of variance (ANOVA) from Table 2, the values of the determination coefficient (R^2^ = 0.9873) and the adjusted determination coefficient (Adjusted R^2^ = 0.9577) both suggested that the actual results could be explained by the obtained model with a high correlation. The P-value of the model was 0.0002, indicating that the model had a highly significant difference at *p* < 0.01. At the same time, the lack-of-fit value was 0.0591, which was higher than 0.05, indicating that the value was negligible to the pure error. In addition, the coefficient estimates influencing the extraction yield (*p* < 0.05), including the linear coefficients (A, B and D), cross coefficients (AB, AD, and CD), and quadratic coefficients (A^2^, B^2^, C^2^, and D^2^), are shown in Table 2, while the other coefficients were not significant (*p* > 0.05).

**Table 2:** ANOVA for response surface quadratic model.

Source	Sum of Squares	DF ^a^	Mean Square	*F*-Value	*p*-Value	Significance ^b^
Model	1366.20	14	97.59	33.32	0.0002	^**^
X_1_	23.12	1	23.12	7.89	0.0308	^*^
X_2_	95.22	1	95.22	32.51	0.0013	^**^
X_3_	10.27	1	10.27	3.51	0.1103	n.s.
X_4_	27.38	1	27.38	9.35	0.0223	^*^
X_1_X_2_	38.03	1	38.03	12.98	0.0113	^*^
X_1_X_3_	6.66	1	6.66	2.27	0.1823	n.s.
X_1_X_4_	77.63	1	77.63	26.51	0.0021	^**^
X_2_X_3_	0.55	1	0.55	0.19	0.6796	n.s.
X_2_X_4_	11.76	1	11.76	4.02	0.0919	n.s.
X_3_X_4_	34.03	1	34.03	11.62	0.0143	^*^
X_1_^2^	1	328.66	112.21	<0.0001	<0.0001	^**^
X_2_^2^	1	452.52	154.50	<0.0001	<0.0001	^**^
X_3_^2^	1	346.23	118.21	<0.0001	<0.0001	^**^
X_4_^2^	1	249.82	85.29	<0.0001	<0.0001	^**^
Residual	17.57	6	2.93			
Lack of fit	13.30	2	6.65	6.22	0.0591	n.s.
Pure error	4.27	4	1.07			
Cor total	1383.77	20				
R^2^	0.9873			Adjusted R^2^	0.9577	

^a^ Degree of freedom; ^b^^*^ *p* < 0.05 significant difference; ^**^ *p* < 0.01 highly significant; n.s. means not significant difference.

**Table 3:** The content of various components in purified polysaccharides.

Polysaccharides	Yield (%)	Uronic Acid (%)	Ginsenside (%)	Protein (%)	Total Carbohydrates (%)
MPPG	41.6 ± 0.09%	10.5 ± 1.77%	-	1.67 ± 0.19%	81.7 ± 3.02%
WPPG	28.5 ± 1.62%	8.5 ± 1.54%	-	1.73 ± 0.36%	84.8 ± 2.98%

“-” means not present.

**Table 4:** Physicochemical properties of purified polysaccharides.

Polysaccharides	Monosaccharide Composition (Molar Ratio)								Peak No.1	Peak No.2
	Man	Rib	Rha	GluA	GalA	Glu	Gal	Arab	Mw (Da)-Area (%)	Mw (Da)-Area (%)
MPPG	3.94	4.55	1.85	1	1.43	141.42	2.7	5.15	1271520-29	411-71
WPPG	3.75	3.42	1.09	-	1	67.6	1.56	1.10	527763-39.1	402-60.9

“-” means not present.

**Table 5:** Minimal inhibitory concentrations (MIC) of purified polysaccharides.

	*S. aureus*	*E. coli*	*B. pumilus*	*B. subtilis*
MPPG (mg/mL)	0.25	0.025	0.01	0.5
WPPG (mg/mL)	0.5	0.05	0.05	0.5

**Table 6:** The code and level of factors selected for the trials.

Independent Variable	Level		
	−1	0	1
Microwave power (W, X_1_)	500	550	600
Liquid-to-solid ratio (mL/g, X_2_)	10: 1	20: 1	30: 1
Extraction time (min, X_3_)	2	4	6
Extraction temperature (°C, X_4_)	50	60	70
